# PARP1 is a versatile factor in the regulation of mRNA stability and decay

**DOI:** 10.1038/s41598-019-39969-7

**Published:** 2019-03-06

**Authors:** Elena A. Matveeva, Lein F. Mathbout, Yvonne N. Fondufe-Mittendorf

**Affiliations:** 1 Department of Molecular and Cellular Biochemistry, University of Kentucky, Lexington, KY USA; 2 College of Medicine, Alfaisal University, Al Maather’, Riyadh, Saudi Arabia

## Abstract

PARP1 is an abundant nuclear protein with many pleiotropic functions involved in epigenetic and transcriptional controls. Abundance of mRNA depends on the balance between synthesis and decay of a particular transcript. PARP1 binds RNA and its depletion results in increased expression of genes involved in nonsense-mediated decay, suggesting that PARP1 might be involved in mRNA stability. This is of interest considering RNA binding proteins play key roles in post-transcriptional processes in all eukaryotes. We tested the direct impact of PARP1 and PARylation on mRNA stability and decay. By measuring the half-lives of two PARP1-mRNA targets we found that the half-lives were significantly decreased in PARP1-depleted cells. PARP1 depletion impacted both the synthesis of nascent mRNA and the stability of mature mRNAs. PARylation impacted the production of nascent mRNA and the stability of mature mRNA, albeit to a lesser extent than PARP1 KD. PARylation enhanced the impact of PARP1 depletion. These studies provide the first direct comparative role of PARP1 and PARylation in RNA stability and decay, adding a new dimension as to how PARP1 regulates gene expression. These studies present a platform to begin to tease out the influence of PARP1 at each step of RNA biogenesis and decay to fine-tune gene expression.

## Introduction

Poly(ADP-ribose) polymerase 1 (PARP1) belongs to a group of proteins known to add single- or poly-ADP ribose to substrates hence their name ADP-ribosyltransferases (ARDTs) or poly(ADP-ribose) synthetases. This family of proteins is quite abundant and the proteins are characterized by having a “PARP signature” motif at the catalytic domain of the active site. This catalytic domain shows 100% conservation among vertebrates and 92% conservation among all species, suggesting a critical role for PARP1 enzymatic activity in cellular function. PARP1 belongs to the sub-group of ARDTs that add poly-ADP ribose moieties to its substrates. PARP1 has been shown to play important roles in different DNA-repair processes, genome integrity, and cell death^1,2^. It also post-translationally modifies several proteins including histones and splicing factors^3,4^, which are critical in gene expression regulation^2,5,6^.

In our previous study, we demonstrated a functional role for PARP1 in the regulation of pre-mRNA splicing^7^. We showed that in addition to its very well-known DNA (chromatin) binding activity, PARP1 also displays both *in vivo* and *in vitro* RNA binding. We also identified the landscape of PARP1-mRNA binding sites^8,9^, placing PARP1 in the group of chromatin binding proteins that also bind RNA. Interestingly, several RNA binding proteins (RBPs) are involved in DNA damage response, and some of these RBPs have been recruited to DNA breaks in a poly (ADP-ribose) (PAR)-dependent manner^10^. Furthermore, recent studies have demonstrated that many chromatin binding proteins also bind different types of RNA such mRNA products to regulate the turnover of these mRNAs (reviewed in^11^). Thus, these proteins modulate gene expression both transcriptionally and post-transcriptionally. In summary, these proteins have been shown to assist in every step of RNA biogenesis, degradation and decay^11,12^.

Our results now put PARP1 in the group of proteins that bind both chromatin and RNA^7,8^. While we have worked extensively on understanding the role of PARP1 in transcription and splicing, the role of PARP1 in mRNA stability is less clear. Our recent studies, however, suggest that PARP1 acts as a genome surveyor. We hypothesize that in the absence of PARP1, irregular splicing products are made, sending a signal for these mis-spliced mRNAs to be degraded. This hypothesis is born from several studies showing that (1) PARP1 modulates HuR^13^, a protein that selectively binds AU-rich elements (AREs) and stabilizes ARE-containing mRNAs and (2) Our previous studies, which showed that knockdown of PARP1 resulted in an increase in gene expression of proteins involved in nonsense-mediated decay (NMD). Interestingly, this increase in the expression of proteins involved in NMD correlated with the accumulation of mis-spliced mRNAs, suggesting a need to degrade mis-spliced RNAs that are detrimental to the cell^8^. RNA degradation pathways, therefore, play important roles in mRNA surveillance machinery to ensure the faithful transmission of genetic information^14,15^. However, many proteins that control RNA stability have not been found, indicating that other players could be important in regulating RNA degradation^16^. We therefore asked the question of whether PARP1 is involved in mRNA stability. This question is not far-fetched, as several studies have shown direct involvement of other PARPs in mRNA stabilization. For instance, PARP13 was shown to influence the stability of TRAIL mRNA^17,18^, while PARP14 influence the stability of the mRNA of tissue factor protein n^19^. Additionally PARP1 was shown to PARylate HuR to modulate the stability of specific mRNAs during pro-inflammation^13^.

Thus, to establish the PARP1 role in mRNA stability and RNAPII transcriptional rate we used a comprehensive approach, combining PARP1 knockdown and PARP1 inhibition with different RNAPII inhibitors: α-amanitin, actinomycin D and DRB. Since the ‘steady-state’ level of transcripts in eukaryotic cells is an outcome of the competition of RNA synthesis and degradation, such combination allowed us to uncouple the effect of physical presence of PARP1 from its catalytic activity on mRNA production and stability/turnover.

## Results

### PARP1 modulates mRNA stability

**Figure 1:**
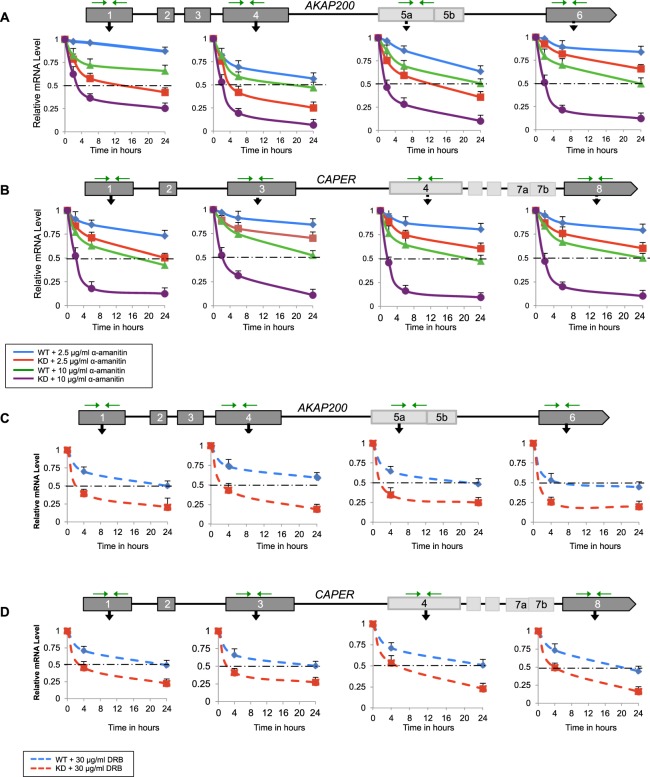
PARP1 depletion destabilizes the mRNA of *AKAP200* and *CAPER*. Half-lives of *AKAP200* and *CAPER* were measured using qRT-PCR in WT and PARP1-KD cells. S2 Drosophila cells were transfected with siCon and siPARP1. The decay rates of total mRNAs were assessed using quantitative RT-PCR in WT and PARP1 KD cells following transcription inhibition by α-amanitin experiments (**A**,**B**) and DRB (**C**,**D**). Mean mRNA half-lives were calculated in WT and PARP1 KD for *AKAP* and *CAPER* transcripts previously shown to be bound by PARP1 at exon/intron boundaries and also alternatively spliced in PARP1 KD cells. Data are represented as mean of three experimental replicates ± SEM. Differences between WT and each experimental condition were measured and results were significant as measured by student T-test, p < 0.05.

The stability of an RNA molecule, as measured by its half-life, is a critical factor in defining timescales for cellular events. It is becoming apparent that mRNA degradation and transcription are intimately linked. Our previous studies showed that depletion of PARP1 resulted in differential alternative splicing (AS) patterns as well as upregulation of genes involved in NMD^8^. We therefore postulate that in the absence of PARP1, mis-splicing of transcripts occurs and eventually these mis-spliced mRNAs will be subjected to NMD, thus reducing their stability. To test this hypothesis and determine the functional consequences of PARP1-mRNA binding in cells, we evaluated the effect of siRNA-mediated knockdown (KD) of PARP1 (Suppl. Fig. S1) on the decay of endogenous known PARP1 targets - *AKAP200* (hence called *AKAP)* and *CAPER* transcripts in *Drosophila* S2 cells. *Drosophila* has only one PARP1 gene, making the understanding of its function relatively easier than in human cells with more than 17 PARPs. Wild type cells (WT) and PARP1 KD S2 cells were treated with α-amanitin to stop transcription by RNA polymerase II and RNA was isolated at 0, 2, 6 and 24 hours post treatment. Then, the decay of *AKAP* and *CAPER* transcripts was measured using real-time polymerase chain reaction (RT-PCR) (Fig. 1). 5S rRNA transcribed by RNAPIII was used as an experimental control to validate the selective transcription inhibition of RNAPII over RNAPIII^20^. Our results show that, indeed, the 5S rRNA levels did not significantly change after α-amanitin treatment and that there was no significant difference between WT and KD cells indicating that 5S rRNA is not a PARP1 target (Suppl. Fig. S2).

For *AKAP* and *CAPER* mRNAs, half-lives were calculated as the time required for each mRNA to decrease to 50% of its initial abundance. α-Amanitin as well as other inhibitors can cause secondary effects on cell physiology^20,21^. Because of this, we treated the cells initially with a lower dose of this compound. Using 2.5 μg/ml α-amanitin, half-life (t_1/2_) was measured at all exons at different time points post treatment. In WT cells, the t_1/2_ as measured from all exons was greater than 30 hours at the *AKAP* gene (Fig. 1A-blue line and Table 1). However, in PARP1 KD, we measured a decrease in mRNA stability, ranging from 5 hours at exon 4 (Ex4) to 15.1 hours at exon 1 (Ex1), while t_1/2_ measured at Ex6 was >30 hours (Fig. 1A-red line and Table 1). These differences were exacerbated when 10 μg/ml of α-amanitin was used. In WT cells, at Ex1, a t_1/2_ > 30 hours was measured, while at the other exons the half-lives ranged from 19.4 hours to 24.7 hours (Fig. 1A-green line and Table 1). In KD cells, a 9–13 fold decline in the mRNA stability was measured (t_1/2_ of 3.8, 2.1, 1.9 and 2 hours for Ex1, 4, 5 and 6 respectively) (Fig. 1A-purple line and Table 1). At the *CAPER* gene, similar results were seen. With 2.5 µg/ml α-amanitin, the half-lives measured in both WT (Fig. 1B-red line and Table 1) and KD (Fig. 1B-blue line and Table 1) cells did not show any significant differences. However, when cells were treated with the higher dose, 10 µg/ml α-amanitin, the changes in stability were quite obvious and drastic. The t_1/2_ measured from all the exons in WT cells were between 17.3 to 24 hours (Fig. 1B-green line and Table 1), while this significantly decreased to ~2 hours in PARP1 knockown cells (Fig. 1B-purple line and Table 1).

**Table 1:** Half-lives in hours of *AKAP200 and CAPER* mRNAs as measured from the different exons after α-amanitin treatment.

Gene/Exon	2.5 mg/ml α-amanitin	2.5 mg/ml α-amanitin	10 mg/ml α-amanitin	10 mg/ml α-amanitin	30 ug/ml DRB	30 ug/ml DRB
***AKAP200***	**WT**	**KD**	**WT**	**KD**	**WT**	**KD**
1	>30	15.1	>30	3.8	24	3.6
4	>30	5	19.4	2.1	>24	3
5	>30	13.1	24.7	1.9	24	2
6	>30	>30	23.7	2	7.3	2.2
***CAPER***						
1	>30	24	17.3	2.3	23.6	3.7
3	>30	>30	24	2	24	3.4
4	>30	>30	24	1.8	24	6.1
8	>30	>30	24	1.9	20	3.9

To confirm that the increase in mRNA decay is a result of PARP1 knockdown and not due to off-target effects caused by α-amanitin, we also inhibited transcription with 5,6-Dichloro-1-β-D-ribofuranosylbenzimidazole (DRB) and actinomycin D. To begin, both WT *Drosophila* S2 and PARP1 KD cells were treated with 30 μg/mL of DRB to stop RNAPII transcription and collected RNAs at time points 0, 4, and 24 hours for maximum effect. We first controlled for the stability of the 5S rRNA and showed a negligible impact on this RNA in both WT and KD cells (Suppl. Fig. S3A). We next measured the half-lives of the mRNA at *AKAP* and *CAPER*. Here too in WT cells, half-lives of 7.3–24 hours and 20–24 hours were measured at the different exons of *AKAP* (Fig. 1C - blue line and Table 1**)** and *CAPER* (Fig. 1D – blue line and Table 1**)** exons respectively. However, in KD cells, the measured half-lives were significantly decreased to 2–3.6 hours and 3.4 to 6.1 hours at the different exonic regions of *AKAP* (Fig. 1C - red line and Table 1**)** and *CAPER* (Fig. 1D - red line and Table 1**)** respectively. Though we observed different half-lives measured from different genic regions of the same gene, we attribute these differences either to the presence of different isoforms or to the location/length of transcript made just before transcription is inhibited. Overall however, *AKAP* and *CAPER* mRNA decay rates were significantly higher in KD cells than in WT cells (Fig. 1C,D) whereas 5S rRNA decay rates were similar in both cell lines (Suppl. Fig. S3). Furthermore, using exon-exon primers to represent mature mRNAs, we obtained very similar results of PARP1’s effect on the *AKAP* mRNA stability (Suppl. Fig. S4). Next, WT and PARP1 KD cells were also treated with 5 μg/mL of actinomycin D, and the RNA was harvested at time points 0, 2, 6 and 24 hours post-treatment. We then measured the mRNA half-lives of our candidate genes *AKAP200* and *CAPER* as well as on 5S rRNA, whose expression will also be targeted by actinomycin D. As a control, we measured the stability of 5S rRNA post-treatment. As expected, there was a slight impact on the stability of the 5S rRNA by actinomycin D treatment in both WT and KD cells (Suppl. Fig. S5). At the *AKAP* gene, WT cells exhibited half-lives ranging from 5.8 hours to 19.8 hours at the various exons (Suppl. Fig. S6A - blue hatched line and Suppl. Table S1) while PARP1 depleted (KD) cells exhibited half-lives ranging from 1.6 hours to 3.9 hours, indicating a dramatic decrease in stability in the PARP1 depleted state (Suppl. Fig. S6A - red hatched line and Suppl. Table S1). Similarly, at the *CAPER* gene, WT cells showed half-lives ranging from 5.8 hours to 20.3 hours (Suppl. Fig. S6B - blue hatched line and Suppl. Table S1), while KD cells measured decreased half-lives of 2 to 4.8 hours (Suppl. Fig. S6B - red hatched line and Suppl. Table S1).

Our results therefore support the hypothesis that PARP1’s presence regulates the stability of *AKAP* and *CAPER* mRNAs. Additionally, the absence of PARP1 promotes degradation of a subset of its target mRNAs in S2 cells. Although mRNA decay is a key determinant of the steady-state concentration for any given mRNA species, relatively little is known on a population level about what factors influence turnover rates and how these rates are integrated into cellular decisions.

### Effect of PARylation inhibition on mRNA stability

**Figure 2:**
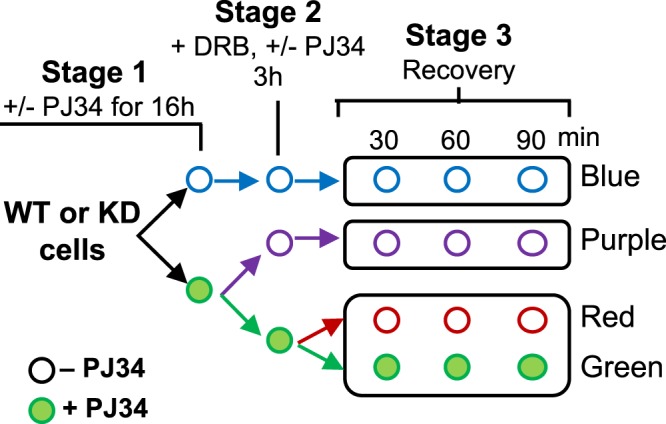
Our experimental design to determine the effect of PARP1 and PARylation on mRNA transcription and stability. Both WT and KD cells were treated overnight with PJ34 to inhibit PARylation (Stage 1). Next, cells were treated with DRB to inhibit transcription (Stage 2). After three hours DRB treatment, cells were washed and transcription was allowed to recover in fresh media without DRB. Cells were then collected at the specified times post DRB-wash (Stage 3).

Several studies have postulated that PARP1 exerts its mRNA stability function through PARylation. For instance, PARP1 PARylates HuR to modulate HuR’s effect on mRNA stability^13^. PARP1 has been shown to also PARylate poly (A) polymerase, resulting in non-poly-adenylated mRNAs that are degraded^22^. However, it is not clear if this function of PARP1 is due to the physical presence of PARP1 or only due to its enzymatic activity. We therefore asked whether the effect of PARP1 on mRNA stability is only through its PARylation activity or if the physical presence of PARP1 plays a direct role on mRNA stability. For this, we carried out a comprehensive study to analyze the impact of PARP1 and PARylation at the different mRNA biogenesis stages in a continuous experimental design (Fig. 2). To begin we measured the effectiveness of PARylation inhibition by PJ34 (Suppl. Fig. S8) and showed that the level of PAR is significantly reduced after inhibition. Next, WT and KD cells were treated with PJ34 (PARylation inhibitor) or vehicle overnight (Stage 1) and then initiating RNAPIIs were transiently inhibited with DRB – Stage 2. Three hours later, DRB was washed off to resume transcription elongation. At each stage, RNA was extracted from the samples and analyzed using quantitative RT-PCR (qRT-PCR) with primers targeting exons to monitor total mRNA. After DRB was washed off, samples were split into two, and RNA transcription recovery was allowed to proceed in the absence or presence of PJ34. The velocity of the resumed transcription wave was again measured using qRT-PCR with primers spanning different regions of the *AKAP* and *CAPER* genes to measure total mRNA. Samples were collected at 30, 60 and 90 min post-DRB to assay the amount of RNA recovered - Stage 3.

**Figure 3:**
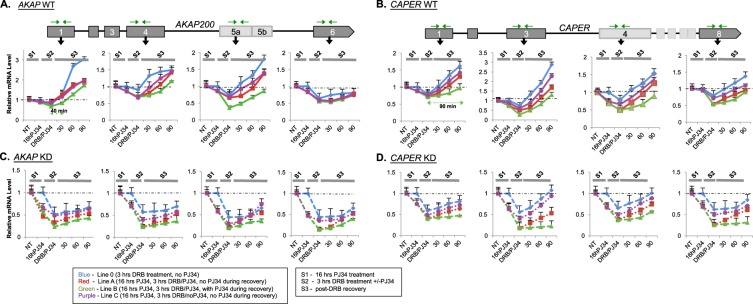
PARP1 and PARylation affects total mRNA levels differentially. Total mRNA levels using exonic primers (green arrows) were measured at each stage of the experiment. WT for and PARP1 KD cells were treated with PJ34 overnight, to inhibit PARylation. Control cells are cells in each condition (WT or KD) that was not treatment with PJ34 at each stage. At the beginning (Stage 1 or S1), mRNA levels were measured from cells. Next, cells were incubated with 30 μg/ml DRB for 3 hrs (Stage 2 or S2); then DRB containing medium was removed, fresh medium was added and transcription was allowed to resume (Stage 3 or S3). At the stage of recovery (S3), levels of total RNA as measured by RT-PCR using exonic primers (green arrows) were determined for *AKAP200* **(A)** and *CAPER* **(B)** and PARP1 KD cells for *AKAP200* **(C)** and *CAPER* **(D)**. mRNA levels were normalized to the values prior to PJ34 treatment sample, which was set to 1. Results are shown as mean ± SEM from three independent experiments. All results were measured in relation to the non-treated cells in each condition (WT or KD) and results were significant as analyzed using student T-test p < 0.05.

All the mRNA samples were quantified and relativized to the level of non-PJ34-treated cells in each condition (WT and KD cells – blue line Fig. 3). In Stage 1, treatment of WT cells with 10 μM PJ34 for 16 hours resulted in a 10–16% decrease in mRNA levels at all exons tested compared to non-treated cells at the *AKAP* gene (Fig. 3A - red, green, purple lines). Similarly, the mRNA levels for *CAPER* decreased by 5–26% in PJ34-treated cells compared to non-treated cells (Fig. 3B-blue line vs. red, green, purple lines**)**. These results suggest that PARylation also affects mRNA stability. However, it was not clear whether the effects of PARP1 on mRNA stability (Fig. 1 and suppl. Fig. S6) were due only to the enzymatic activity (PARylation) of PARP1 or to physical presence of PARP1. To answer this question, we measured the mRNA stability of PJ34-treated KD cells. Here, we detected a 40–50% and a 25–37% decline in mRNA stability at the various *AKAP* exons (Fig. 3C - vs. red, green, purple lines) and *CAPER* exons (Fig. 3C - vs. red, green, purple lines**)** respectively. Since the effect of PJ34 in the presence of PARP1 was not as strong as in its absence, we believe the result seen here is due to an additive effect of PARylation inhibition onto the impact of PARP1 depletion. These results suggest that the impact of PARP1 on total mRNA stability (transcription and production) is stronger than depleting PARylation alone. Our interpretation is based on the finding that inhibition of PARylation completely wiped out the PAR levels while in PARP1 KD cells with 75% PARP1 depletion, the levels of PAR decreased only by about 40 (Suppl. Fig. S8**)**.

In Stage 2, transcription was inhibited with DRB in the absence or presence of PJ34. This stage represents the effect of PARP1/PARylation on mRNA stability in the absence of mRNA production. Here too, mRNA levels were relativized to the levels in cells that were never treated with PJ34 (Fig. 3 - Blue line). In WT cells, PJ34 seemed to have very little effect on mRNA stability at Exons 1 of *AKAP* (Fig. 3A – blue line vs. red, green, purple lines**)** and *CAPER* (Fig. 3B - blue line vs. red, green, purple lines and Table 2), respectively. However, the PARylation clearly impacted the stability of mRNAs downstream of exon 1 at both genes. This impact of PARylation on mRNA stability at all exons tested is magnified in KD cells. After transcription inhibition, KD cells treated with PJ34 showed an additional 5–30% decrease in mRNA stability at Exon 1 compared to cells non-PJ34 treated. And the measured mRNA stability at the other exons also confirmed this additive effect of PARylation on the stronger effect from PARP1 depletion (Fig. 3C,D - blue line vs. red, green, purple lines and Table 2).

**Table 2:** Time (min) till complete recovery of mRNA level for *AKAP200* and *CAPER* in WT and PARP1 KD *Drosophil*a S2 cells. (Exon primers).

WT	*AKAP200*				*CAPER*			
	Ex1	Ex4	Ex5	Ex6	Ex1	Ex3	Ex4	Ex8
Blue line	13	7	22	375	25	13	23	31
Red line	10	53	64	138	53	51	66	99
Green line	40	75	113	147	90	71	103	104
Purple line	13	35	42	196	40	36	48	60
KD	***AKAP200***				***CAPER***			
	**Ex1**	**Ex4**	**Ex5**	**Ex6**	**Ex1**	**Ex3**	**Ex4**	**Ex8**
Blue line	213	190	148	148	193	80	90	115
Red line	500	173	333	212	281	215	142	196
Green line	588	491	1228	217	596	1330	176	1022
Purple line	226	240	123	212	217	98	118	157

The difference in the effect of PARP1/PARylation on mRNA stability is clearly seen in the comparison of the cells treated with DRB in the absence or presence of PJ34 (Fig. 3A–D -blue lines vs. purple lines). In fact, the cells that were previously treated with PJ34 and then had the drug removed during DRB treatment (Stage 2) showed a slight increase in mRNA levels during recovery (Stage 3; Fig. 3A–D, blue line vs. purple lines). These results support our idea that the effect of PARP1 on mRNA stability is stronger and more direct while the effect of PARylation is not as robust and possibly indirect. This idea is supported also by directly comparing the mRNA levels in WT and PJ34-treated-WT cells. These results again demonstrate that PARP1, directly and indirectly through PARylation of mRNA stability factors^8,13,22,23^, plays a role in mRNA stability.

Changes in the rate of synthesis and/or the rate of degradation of either of the transcripts would give rise to the changes in the relative magnitudes of these transcripts. To test whether PARP1 influences both degradation and transcription in this continuous model, WT and PARP1 knockdown cells were treated for 3 hours with DRB in the presence or absence of PJ34 and new transcription was allowed to start in the absence or presence of PJ34 (Stage 3). Samples were harvested at 0, 30, 60 or 90 min post DRB treatment and RNA levels were measured. Since the levels of mRNA in WT and PARP1 KD cells after DRB treatment were different at time point 0 for each experiment, mRNA recovery concentration was quantified relative to this time point for each experiment. Additionally, we normalized the starting mRNA concentration to 1 - stage 0 (the stage before any treatment), to determine the level/speed of recovery. In WT cells not treated with PJ34, recovery at Ex1 was about 13 mins for *AKAP* (Fig. 3A – blue line and Table 2) while WT cells treated throughout all the experimental conditions (stages), exhibited recovery that was delayed to about 40 mins (Fig. 3A – green line). Similarly, at Ex1 of *CAPER*, recovery occurred in about 25 mins (blue line Fig. 3B) while cells with constant PARylation inhibition (green line- Fig. 3B) showed a delay of about 90 minutes. In line with our idea that PARP1’s presence is stronger, KD cells never recovered in the absence or presence of PJ34 (Fig. 3C,D). Finally a direct comparison between WT and KD cells is represented in Suppl. Fig. S7. Taken together, these results strongly suggest the influence of PARP1 in transcription and decay for the tested transcripts.

### The effect of PARP1 on nascent mRNA production is stronger than the effect of its enzymatic activity

To obtain a more direct measurement of the effect of PARP1/PARylation on pre-mRNA production, we analyzed the accumulation of nascent pre-mRNA at different exons using exon-intron primers. For this, just as above, pre-mRNA was measured in our experimental set-up using primer pairs targeting specific exon-intron junctions. The goal of these experiments was to first determine the effect of the physical presence of PARP1 depletion on RNAPII activity in the context of transcript production and elongation. To do this, we compared pre-mRNA accumulation between WT and KD cells. The second goal was to assess the effect of PARylation on transcript production and transcriptional elongation. For this, we compared the pre-mRNA accumulation between PJ34-treated WT and KD cells at the different stages of the experiment setup compared with the non-treated cells under similar conditions (Fig. 2). In all cases, the levels of pre-mRNA before any treatment in both WT and KD cells were normalized to 1 and the pre-mRNA levels at each time point was relativized to this level.

**Figure 4:**
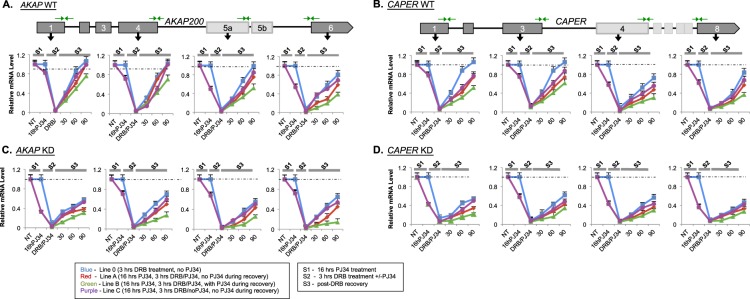
Depletion of PARP1/PARylation affects transcription and PolII recovery rate as measured by nascent mRNA levels. Time-course of transcription elongation for *AKAP* and *CAPER* genes post-DRB treatment. WT and PARP1 KD cells were incubated with DRB for 3 hrs to halt transcription. Then post-DRB wash, transcription was allowed to start and continue in a synchronized manner. Levels of pre-mRNA as measured by RT-PCR using exon-intron junction primers (green arrows) were determined for *AKAP200* **(A)** and *CAPER* **(B)** and PARP1 KD cells for *AKAP200* **(C)** and *CAPER* **(D)**. Pre-mRNA levels were normalized to the values prior of the prior-DRB treatment sample, which was set to 1. Results are shown as mean ± SEM from three independent experiments. Differences between NT and each experimental condition were considered significant according to student T-test p < 0.05.

To begin, pre-mRNA levels were measured after cells were exposed to 16 hours of PJ34 (Stage 1). Compared to non-exposed PJ34 WT and KD cells (blue line – Fig. 4), exposed cells showed a decrease in pre-mRNA levels (Fig. 4 - red, green and purple lines), consistent with the known effect of PARylation on increased gene expression. Next, cells were treated with DRB in the absence or presence of PJ34 – stage 2. Indeed, DRB halted transcription in both WT and KD cells as the measured levels of pre-mRNA dropped to near zero values (Fig. 4). Finally, DRB was washed off and transcription was allowed to continue post-DRB wash (Stage 3). Transcription in cells was considered fully recovered when the pre-mRNA levels reached 1 (initial pre-mRNA level before any treatment – Stage 1). As can be seen from the pre-mRNA analysis, transcription of the proximal region (exon 1 for both *AKAP* and *CAPER*) resumed transcription fully within 90 min after DRB removal in WT cells In contrast, transcription of the distal regions of the same gene (exon 6 of *AKAP* and exon 8 of *CAPER*) downstream from the TSS did not show recovery within the time frame of our measurements (90 minutes after drug release), consistent with the genomic distance between the two regions (Fig. 4A,B respectively, and Table 3). We next determined the impact of PARylation on nascent mRNA production. Not surprisingly, we found and confirmed previous results on the impact of PARylation on transcription. Generally, the effect of PJ34 is as follows: cells not exposed to PJ34 recovered the fastest (blue line) followed by cells PJ34-exposed in one of the three experimental stages (purple line). Cells PJ34-exposed in two of the three experimental stages (red line) recover next, followed by cells in which PARylation was inhibited in all of the experimental stages (green line) – Fig. 4A for *AKAP*. These results were recapitulated at the *CAPER* gene (Fig. 4B). In KD cells, PARylation inhibition in cells augmented the impact of PARP1 depletion on transcription inhibition, with a much stronger depletion on pre-mRNA levels in KD cells compared to WT cells. Additionally, KD cells failed to fully recover transcriptionally post-DRB wash as measured within the 90 mins of our experimental setting for all exons of both *AKAP* (Fig. 4C) and *CAPER* genes (Fig. 4D). Furthermore, the inhibition of PARylation in these KD cells, though exacerbating the impact of PARP1 depletion, followed a similar profile to the effect in WT cells. Specifically, WT and KD cells treated with PJ34 throughout all three experimental stages (Fig. 2) had the greatest impact on mRNA production at both *AKAP* and *CAPER* genes in both cells, respectively (Fig. 4C,D - green line, Table 3), followed by cells treated at two of the three stages (red line), cells treated at only one stage (purple line), and cells not treated at all with PJ34 (blue line). In summary, PARylation augmented and enhanced the negative effect of PARP1 depletion on mRNA stability. We therefore interpret these results to suggest that the effect of the physical presence of PARP1 on mRNA production is stronger than the enzymatic activity. However, the combined effects of physical presence and enzymatic activity complement each other to drive normal mRNA production.

**Table 3:** Time (min) till complete recovery of mRNA level for *AKAP200* and *CAPER* in WT and PARP1 KD *Drosophila* S2 cells. (Exon/Intron primers).

WT	*AKAP200*				*CAPER*			
	Ex1	Ex4	Ex5	Ex6	Ex1	Ex3	Ex4	Ex8
Blue line	90	90	90	116	90	90	131.7	101.4
Red line	90.3	89.2	114	129	113.6	123.9	177.5	129.6
Green line	118	122.3	165	183.8	159.7	145.4	359.6	206.8
Purple line	91.4	90	102	139.3	116.8	105.2	170.3	117.6
**KD**	***AKAP200***				***CAPER***			
	**Ex1**	**Ex4**	**Ex5**	**Ex6**	**Ex1**	**Ex3**	**Ex4**	**Ex8**
Blue line	186.7	137.4	165	148.3	243.5	146.6	173.4	195.6
Red line	458.4	178	162.7	167.6	298.2	210.4	247.4	375.1
Green line	319	327.1	227.8	958.7	442	198.6	278.1	287.3
Purple line	183.3	142	164	202	171.5	186	292.1	207.8

To summarize, both *AKAP* and *CAPER* mRNAs showed decreased stability following PARP1 KD (Fig. 3 and suppl. Fig. S7) as well as decreased levels of pre-mRNAs, indicating reduced transcription (Fig. 4). Based on these studies, we are observing two aspects of PARP1 in RNA biogenesis. (1) PARP1 plays a role in RNA stability. This is clearly shown in the experiments where no transcription is taking place and we are simply measuring mRNA turn-over in the cell. (2) PARP1 is important for RNA transcription. In recovery experiments, the levels of mRNA production (measured by exon-intron primers) show that in the presence of PARP1, transcription is occurring normally, whereas the rate of transcription in KD cells is hampered. In summary, these results show that PARP1 plays a significant role in mRNA biogenesis and stability.

**Figure 5:**
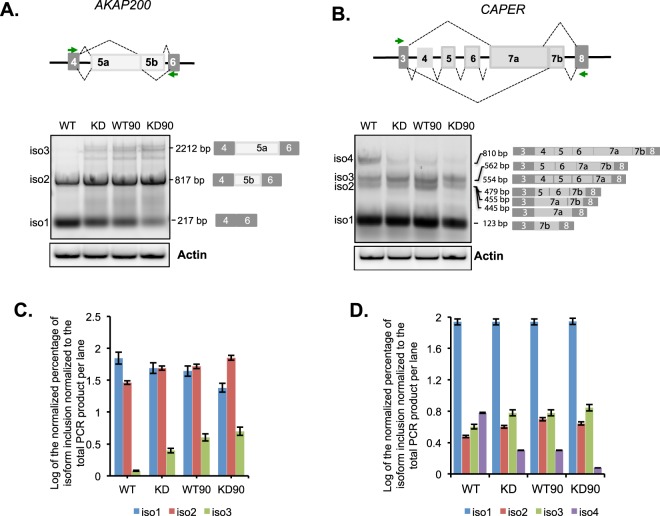
PARP1-mediated coupling of RNAPII elongation and splicing kinetics is impaired in PARPI depletion. PCR products with exon junction spanning primers validate splicing changes in *AKAP200* (**A**) and *CAPER* (**B**). Agarose gel images show the difference in splicing products between wildtype-non-treated (WT) and PARP1 knockdown (KD) cells before DRB treatment and at 90 min post-DRB recovery time point (WT90 and KD90 respectively). Actin was used as a PARP1-non-target control. The intensity of all bands per lane was quantified and the total band quantification was set to 100%. The relative amount of each band per lane was calculated as a percentage of the total band intensities per lane. The log values of the relative amount of each band to the total PCR product per lane for *AKAP* (**C**) and *CAPER* (**D**). Log values were plotted for easier visualization of the differences.

Finally, we asked whether the cells undergoing recovery have differences in splicing. According to the kinetic model of co-transcriptional splicing, any factor that affects RNAPII elongation should have a splicing pattern similar to the splicing pattern seen after DRB treatment (since DRB affects RNAPII elongation). We therefore reviewed the splicing profile after 90 min of recovery post-DRB treatment. We show that the splicing pattern in WT cells post-DRB at both the *AKAP* and *CAPER* genes mirrors the pattern seen after PARP1 KD cells and all three differ from the pattern seen at non-DRB treated WT cells (Fig. 5). Our results confirm that PARP1 plays a role not only in mRNA stability, but also in AS decisions.

## Discussion

PARP1 is typically known for its chromatin binding activities. We recently showed that PARP1 binds RNA and modulates co-translational splicing, prompting us to ask if PARP1 has any effect on the post-transcriptional fate of mRNA. In this study, we took a comprehensive approach to determine whether PARP1 and/or PARylation affect mRNA post-transcriptionally, specifically mRNA stability. We used three different transcription inhibitors and demonstrated that in the absence of PARP1, the stability of *AKAP* and *CAPER* mRNAs were significantly reduced. By measuring the ‘steady state’ mRNA levels (which is dependent on the competition between RNA synthesis and degradation), we show that while PARylation affected the steady state RNA level, the effect of PARP1 depletion was far greater. We also show that the impact of PARylation on nascent mRNA synthesis was not as strong as the effect of PARP1 depletion. These findings suggest that compared to PARylation, PARP1 has a considerable impact on mRNA stability and turnover.

PARP1 may be influencing mRNA stability in several ways. It could aid directly in mRNA stability or it could recruit proteins that aid in mRNA stabilization, thus preventing mRNA degradation. On the other hand, PARP1 indirectly through PARylation, could modulate the activity of proteins important for mRNA stability. The results as presented in our study show that the effect of PARP1 depletion on mRNA stability is strongly related to the direct effect of PARP1. We posit this direct regulation of PARP1 because the mRNAs of *AKAP* and *CAPER* used in this study are targets of PARP1-mediated AS. And hypothesize that PARP1 depletion leads to their mis-splicing and eventual degradation. By using α-amanitin to inhibit transcription, half-life analyses showed that PARP1 depletion resulted in the decay of *AKAP* and *CAPER* mRNAs (Fig. 1). We further confirmed this effect of PARP1, using two other transcription inhibitors, DRB and actinomycin D (Fig. 1C,D and suppl. S6 respectively). We thus provide direct evidence for the role of PARP1 in regulating mRNA stability.

Measuring decay at exons, does not distinguish pre-mRNAs from mature mRNAs. We posit that the decays measured in Fig. 1 and suppl. Fig. S6, represent ‘steady-state’ levels (mRNAs at all stages – production, processing and decay). Indeed different steps of pre-mRNA processing, from 5′ capping, splicing, polyadenylation to nuclear export, are tightly co-regulated and coordinated with transcription, translation and mRNA decay. How PARP1 mediates these processes is still unknown. Interestingly, several studies have shown that PARP1 acts in most of the above steps, to fine-tune gene expression. For instance, PARP1 binds promoters and enhancers to regulate transcription initiation^1,7,24–27^. It does so through a variety of mechanisms including altering chromatin accessibility^24,25,28–30^, acting as a co-regulator of transcription factors^31^ and regulating DNA and histone methylation patterns^32–37^ to drive specific gene expression patterns^1,7,24,29,33,38–43^. Next mRNA is processed through cotranscriptional splicing. PARP1 binds hnRNPs^4,44,45^, modulating their activity, to alter splicing decision^4,45^. We further showed that PARP1 influences splicing decisions by modulating the chromatin structure^7^. In support of the role of PARP1 in different stages of RNA biogenesis PARP1 PARylates proteins are involved in RNA metabolism^23^, mRNA processing^46^, mRNA metabolism, RNA splicing, RNA export and protein synthesis^23,47^.

We recently illustrated that not only does PARP1 influence AS through its enzymatic activity, but also through its ability to modify chromatin structure. Indeed, the alternative splicing events affected by PARP1 depletion were different from those affected by PARylation. These results suggest a direct effect by PARP1 while that of PARylation maybe indirect in PARylating splicing factors. We showed that PARP1 creates and/or maintains a chromatin structure that alters the rate of RNAPII elongation, important for differential splicing outcomes^7^. We demonstrated further that PARP1 binds to RNA, bridging RNA to chromatin and recruit splicing factors to specific splice sites. Thus, while PARylation is important in modulating the activity of splicing factors, PARP1’s physical presence aids creates/maintains important regulatory structures critical for splicing. We also found in the present study that both PARylation inhibition and PARP1 depletion affected the stability of *AKAP* and *CAPER* mRNAs. However, the mRNA stability effect of PARP1 depletion was stronger than that caused by PARylation. We see this both in its effect on total mRNA (Fig. 3 and suppl. Fig. S7) as well as on nascent mRNA production (Fig. 4).

RNA binding proteins act at several steps in mRNA biogenesis to fine-tune gene expression post-transcriptionally^48^. Our measurement of total RNA includes RNAs at different stages of biogenesis – transcriptional as well as post-transcriptional stages. In this study we measured transcription initiation after recovery but not the effect of PARP1 on different stages of mRNA processing such as capping, polyadenylation or translation. However, it is possible that our results on the decay of total RNA, could involve PARP1’s effect on all of these stages. PARP1 is known to PARylate poly (A) polymerase, consequently inhibiting its activity to polyadenylate mRNAs resulting in their subsequent degradation^22^. In addition, PARP1 PARylates ribosomal proteins, thus impacting protein translation^47^. These studies are consistent with our finding that several ribosomal proteins are part of the PARP1 interactome^7^. Since all these studies point to a role of PARP1 in mRNA decay, this would indicate another component in PARP1’s regulation of gene expression, by maintaining a careful balance between transcript production and the mRNA decay.

There are many degradation pathways in the cells and it is not clear which pathway is involved in PARP1-mediated mRNA degradation. PARP1 KD resulted in increased expression of genes involved in NMD^8^, possibly linking PARP1 to the NMD pathway. PARP1 could be part of a complex in this pathway or proteins in this pathway are PARylated. However, future studies will be needed to determine this. This is of interest as the stability of specific transcripts is determined, in part, by the binding of RBPs to cis-acting sequences such as AREs connecting these target mRNAs to core degradation pathways^49,50^. In support of PARP1’s role in mRNA stability, PARylation of HuR, a protein that binds to AREs, increases mRNA stability^13^. Though we do not directly test the mechanism of PARP1 in the stability of *AKAP* and *CAPER mRNAs*, we postulate that it acts through the NMD surveillance pathway. This idea is born from our finding that PARP1 KD resulted in (1) the generation of alternative isoforms of these genes, and (2) increased transcription of genes involved in NMD^8^. This is an attractive idea considering NMD has been shown to degrade unproductive spliced or aberrant transcripts in several organisms including *C*. *elegans*^51^, *S*. *cerevisiae*^52,53^, and *Drosophila*^54^. Hence, NMD is postulated to act as a general quality control mechanism for defective or suboptimal splicing. These degradation pathways thus serve as a surveillance way to determine the quality of the mRNA produced. While it is exciting to determine how PARP1 regulates mRNA stability and decay, the challenge ahead will be to determine the mechanism by which it does so.

In summary, we have systematically characterized the impact of PARP1 and PARylation on mRNA stability. Our results offer new insights and provide a valuable resource to begin to tease out mechanistically, the role of PARP1 in mRNA decay and biogenesis.

## Materials and Methods

### S2 cell culture and siRNA mediated knockdown

*Drosophila melanogaster* S2 cells (obtained from Thermo Fisher Scientific, Waltham, MA) were cultured in Schneider’s *Drosophila* medium (Life Technologies, Austin, TX) supplemented with 10% heat activated fetal bovine serum (Sigma, St. Louis), 100 U/ml penicillin and 100 µg/ml streptomycin at 25 °C. All experimental samples and controls were growth time and cell-density matched. siRNA mediated PARP1 knockdown was done exactly as described in Matveeva *et al*.^7^. Depletion of PARP1 was confirmed by Western blot and quantitative PCR with primers 1–4 (Suppl. Table S2).

### S2 cells extract preparation

*Drosophila melanogaster* S2 cells 10^7^ were resuspended in 0.5 ml of RIPA buffer with fresh added inhibitors: 1 mM PMSF (Phenylmethylsulfonyl fluoride, Sigma, St Louis, MO, #10837091001), 1xProtease inhibitor cocktail (EpiGentek, Farmingdale, NY, #R-1101), 10 mM 3-MBZ (3-Methoxybenzylamine, Sigma, #159891) and 0.5 mM BZA (Benzylamine, Sigma, #185701), incubated for 30 min on ice with mild vortexing, and then sonicated for 6 cycles (10 sec on/10 sec off (Bioruptor300, Diagenode, Denville, MJ) and spun down at 13000 rpm for 10 min. Supernatant was used for Western blot analyses.

### Western blots

Western blots were performed per standard protocol and input dilutions were used as a quantitative indication of signal linearity. Protein samples were re-suspended in SDS loading buffer and electrophoresed on a 10% SDS-PAGE gel, blotted to PVDF membrane (Thermo Scientific, Rockford IL) and sequentially probed with primary antibodies for PARP1 and Actin to demonstrate PARP1 knockdown, or for PAR and Actin for visualization of PAR levels. Western blot-based detection was performed using alkaline phosphatase-coupled secondary antibodies (Sigma, St Louis, MO) with Amersham ECF substrate for visualization (GE Healthcare, Waukesha, WI) and images were obtained using Typhoon FLA 9500 (GE Healthcare, Piccataway, NJ). ImageQuant TL software was used to quantify protein signals.

### Antibodies for Western blot analysis

Primary: PARP1 C terminal, rabbit polyclonal (#39561, Active Motif, Carlsbad, CA 92008); Actin, mouse monoclonal (MA1-744, ThermoFisher Scientific, Waltham, MA 02451); PAR rabbit polyclonal (#4336-BPC-100, Trevigen, Gaithersburg, MD 20877). Secondary: anti-rabbit (# A3687) and anti-mouse (#A3562) IgG (whole molecule) Alkaline Phosphatase antibody (Sigma, St Louis, MO 63146).

### PAR assay in cell extract

PAR level in *Drosophila melanogaster* S2 cells extracts were measured using HT PARP *in-vivo* Pharmacodynamic Assay II kit as per manufactured protocol (#4520-096-K, Trevigen, Gaithersburg, MD 20877).

### Treatment with RNAPII inhibitors for mRNA stability assay

To analyze the decay rate of *AKAP* and *CAPER* mRNA in S2 cells, 3 × 10^6^ cells (Wild type [WT] and PARP1 knockdown [KD]) were incubated with 2.5 or 10 μg/ml α-amanitin, 5 μg/ml actinomycin D and 30 μg/ml of DRB. A total of 1 × 10^6^ cells were removed at each time point over 4 hour (DRB) or 24 hours (α-amanitin and actinomycin D) for RNA isolation and cDNA synthesis. Untreated 1 × 10^6^ of NT or KD cells were used as time zero point control.

### mRNA recovery rate assay

To observe newly transcribed mRNA 3 × 10^6^ cells, WT and PARP1 KD were incubated with 30 μg/ml 5,6-Dichlorobenzimidizole (DRB) for 3 hours. After DRB removal, 1 × 10^6^ cells were collected at several time points (0, 30, 60 and 90 min) and were subject to RNA purification and cDNA synthesis. To uncouple the effect of PARP1 physical presence from its physiological activity on RNAPII recovery rate, cells were treated with 10 μM PJ34 (PARP1 inhibitor) for 10 hours before DRB treatment.

### RNA isolation and cDNA synthesis

RNA was isolated using RNeasy Plus Mini kit from Qiagen (Gaithersburg, MD) and used for reverse-transcription reaction with iScript reverse transcriptase (Bio-RaD Laboratories, Hercules, CA), 1 µg of RNA per reaction. The resultant cDNAs were used in PCR (S1000 Thermal Cycler, Bio-Rad) with the indicated primer sets (Suppl. Table S2, primers 5–20).

### Analysis and quantitation by PCR

All sets of primers were designed using Integrated DNA Technologies Primer Tools. Quantitative PCR analysis in real time was performed by using CFX96 Real-Time System (Bio-Rad) with TaqDNA polymerase (MB042-EUT-10000, Syd Labs, Natick, MA) and EvaGreen dye (Biotium). Reactions were performed at 25 µl and cycling was for 4 min at 94 °C, followed by 40 cycles of 45 s at 94 °C, 30 s at 60 °C and 60 s at 72 °C. For quality control purposes, melting curves for all samples were acquired (10 s at 95 °C and 60 s at 60 °C). Experiments were conducted in triplicate with at least three biological replicates. For primer sequences see Suppl. Table S3.

### Isoform expression in pre and post DRB treated cells

Total RNAs were isolated, reverse transcribed as described at RNA isolation and cDNA synthesis. The resultant cDNAs were amplified on S1000 Thermal Cycler (Bio-Rad) with primers 7,12,15 and 20 (Suppl. Table 2). PCR cycling parameters were as follows: 94 °C for 4 min; 30 cycles of 45 s at 94 °C, 30 s at 60 °C, and 60 s at 72 °C. PCR products were analyzed on 3.3% NuSieve agarose gel (Lonza, Rockland, ME, 04841) with GelStar Nucleic Acid Gel Stain (Lonza, Rockland, ME, 04841) and visualized with Typhoon FLA 9500 (GE Healthcare, Piccataway, NJ 08854). ImageQuant TL software was used to quantify band signals to calculate relative isoform expression. The mean value of the intensity of each band is shown as a percentage of the total PCR product, which was normalized to 100%. Splice isoforms were confirmed by cloning the products from PCR analyses using PCR Cloning Kit (NEB) according to manufacturer’s protocol and sequenced by Eurofins Scientific.

### Genes of interest

*AKAP200* (Flybase ID: FBgn0027932, symbol: CG13388).*CAPER* (Flybase ID: FBgn0031883, symbol: CG11266).

## Supplementary information

Supplementary Figures and Tables

## Data Availability

All data needed to evaluate the conclusions in the paper are present in the paper and/or the Supplementary Materials. Additional data related to this paper may be requested from the authors.

## References

[1] Kraus WL (2008). Transcriptional control by PARP-1: chromatin modulation, enhancer-binding, coregulation, and insulation. Curr Opin Cell Biol.

[2] Krishnakumar R, Kraus WL (2010). The PARP side of the nucleus: molecular actions, physiological outcomes, and clinical targets. Mol Cell.

[3] Caruso LB (2018). Poly(ADP-ribose) Polymerase 1, PARP1, modifies EZH2 and inhibits EZH2 histone methyltransferase activity after DNA damage. Oncotarget.

[4] Ji Y, Tulin AV (2013). Post-transcriptional regulation by poly(ADP-ribosyl)ation of the RNA-binding proteins. Int J Mol Sci.

[5] Frizzell KM (2009). Global analysis of transcriptional regulation by poly(ADP-ribose) polymerase-1 and poly(ADP-ribose) glycohydrolase in MCF-7 human breast cancer cells. J Biol Chem.

[6] Wacker DA, Frizzell KM, Zhang T, Kraus WL (2007). Regulation of chromatin structure and chromatin-dependent transcription by poly(ADP-ribose) polymerase-1: possible targets for drug-based therapies. Subcell Biochem.

[7] Matveeva E (2016). Involvement of PARP1 in the regulation of alternative splicing. Cell Discov.

[8] Melikishvili M, Chariker JH, Rouchka EC, Fondufe-Mittendorf YN (2017). Transcriptome-wide identification of the RNA-binding landscape of the chromatin-associated protein PARP1 reveals functions in RNA biogenesis. Cell Discov.

[9] Melikishvili M, Matveeva E, Fondufe-Mittendorf Y (2017). Methodology to Identify Poly-ADP-Ribose Polymerase 1 (PARP1)-mRNA Targets by PAR-CLiP. Methods Mol Biol.

[10] Kai M (2016). Roles of RNA-Binding Proteins in DNA Damage Response. Int J Mol Sci.

[11] Hudson WH, Ortlund EA (2014). The structure, function and evolution of proteins that bind DNA and RNA. Nat Rev Mol Cell Biol.

[12] G Hendrickson D, Kelley DR, Tenen D, Bernstein B, Rinn JL (2016). Widespread RNA binding by chromatin-associated proteins. Genome Biol.

[13] Ke Y (2017). PARP1 promotes gene expression at the post-transcriptiona level by modulating the RNA-binding protein HuR. Nat Commun.

[14] Akamatsu W (2005). The RNA-binding protein HuD regulates neuronal cell identity and maturation. Proc Natl Acad Sci USA.

[15] Tani H (2012). Genome-wide determination of RNA stability reveals hundreds of short-lived noncoding transcripts in mammals. Genome Res.

[16] Hasan A, Cotobal C, Duncan CD, Mata J (2014). Systematic analysis of the role of RNA-binding proteins in the regulation of RNA stability. PLoS Genet.

[17] Todorova T, Bock FJ, Chang P (2014). PARP13 regulates cellular mRNA post-transcriptionally and functions as a pro-apoptotic factor by destabilizing TRAILR4 transcript. Nat Commun.

[18] Todorova T, Bock FJ, Chang P (2015). Poly(ADP-ribose) polymerase-13 and RNA regulation in immunity and cancer. Trends Mol Med.

[19] Iqbal MB (2014). PARP-14 combines with tristetraprolin in the selective posttranscriptional control of macrophage tissue factor expression. Blood.

[20] Bensaude O (2011). Inhibiting eukaryotic transcription: Which compound to choose? How to evaluate its activity?. Transcription.

[21] Ross J (1995). mRNA stability in mammalian cells. Microbiol Rev.

[22] Di Giammartino DC, Shi Y, Manley JL (2013). PARP1 represses PAP and inhibits polyadenylation during heat shock. Mol Cell.

[23] Jungmichel S (2013). Proteome-wide identification of poly(ADP-Ribosyl)ation targets in different genotoxic stress responses. Mol Cell.

[24] Krishnakumar R (2008). Reciprocal binding of PARP-1 and histone H1 at promoters specifies transcriptional outcomes. Science.

[25] Petesch SJ, Lis JT (2012). Overcoming the nucleosome barrier during transcript elongation. Trends Genet.

[26] Wisnik E, Ploszaj T, Robaszkiewicz A (2017). Downregulation of PARP1 transcription by promoter-associated E2F4-RBL2-HDAC1-BRM complex contributes to repression of pluripotency stem cell factors in human monocytes. Sci Rep.

[27] Zhao H (2015). PARP1- and CTCF-Mediated Interactions between Active and Repressed Chromatin at the Lamina Promote Oscillating Transcription. Mol Cell.

[28] Cohen-Armon M (2007). DNA-independent PARP-1 activation by phosphorylated ERK2 increases Elk1 activity: a link to histone acetylation. Mol Cell.

[29] Krishnakumar R, Kraus WL (2010). PARP-1 regulates chromatin structure and transcription through a KDM5B-dependent pathway. Mol Cell.

[30] Martinez-Zamudio R, Ha HC (2012). Histone ADP-ribosylation facilitates gene transcription by directly remodeling nucleosomes. Mol Cell Biol.

[31] Hassa PO, Covic M, Hasan S, Imhof R, Hottiger MO (2001). The enzymatic and DNA binding activity of PARP-1 are not required for NF-kappa B coactivator function. J Biol Chem.

[32] Althaus FR (2005). Poly(ADP-ribose): a co-regulator of DNA methylation?. Oncogene.

[33] Bi FF, Li D, Yang Q (2013). Promoter hypomethylation, especially around the E26 transformation-specific motif, and increased expression of poly (ADP-ribose) polymerase 1 in BRCA-mutated serous ovarian cancer. BMC Cancer.

[34] Ciccarone F (2012). Poly(ADP-ribosyl)ation acts in the DNA demethylation of mouse primordial germ cells also with DNA damage-independent roles. PLoS One.

[35] Hashimoto H, Vertino PM, Cheng X (2010). Molecular coupling of DNA methylation and histone methylation. Epigenomics.

[36] Nalabothula N (2015). Genome-Wide Profiling of PARP1 Reveals an Interplay with Gene Regulatory Regions and DNA Methylation. PLoS One.

[37] Zampieri M (2012). ADP-ribose polymers localized on Ctcf-Parp1-Dnmt1 complex prevent methylation of Ctcf target sites. Biochem J.

[38] Kraus WL, Lis JT (2003). PARP goes transcription. Cell.

[39] Ogino H (2007). Loss of Parp-1 affects gene expression profile in a genome-wide manner in ES cells and liver cells. BMC Genomics.

[40] Petesch SJ, Lis JT (2012). Activator-induced spread of poly(ADP-ribose) polymerase promotes nucleosome loss at Hsp70. Mol Cell.

[41] Tulin A, Spradling A (2003). Chromatin loosening by poly(ADP)-ribose polymerase (PARP) at Drosophila puff loci. Science.

[42] Verdone L (2015). Poly(ADP-Ribosyl)ation Affects Histone Acetylation and Transcription. PLoS One.

[43] Zhang T (2012). Regulation of poly(ADP-ribose) polymerase-1-dependent gene expression through promoter-directed recruitment of a nuclear NAD+ synthase. J Biol Chem.

[44] Gagne JP, Hunter JM, Labrecque B, Chabot B, Poirier GG (2003). A proteomic approach to the identification of heterogeneous nuclear ribonucleoproteins as a new family of poly(ADP-ribose)-binding proteins. Biochem J.

[45] Ji Y, Tulin AV (2009). Poly(ADP-ribosyl)ation of heterogeneous nuclear ribonucleoproteins modulates splicing. Nucleic Acids Res.

[46] Zhang Y, Wang J, Ding M, Yu Y (2013). Site-specific characterization of the Asp- and Glu-ADP-ribosylated proteome. Nat Methods.

[47] Isabelle M (2010). Investigation of PARP-1, PARP-2, and PARG interactomes by affinity-purification mass spectrometry. Proteome Sci.

[48] Glisovic T, Bachorik JL, Yong J, Dreyfuss G (2008). RNA-binding proteins and post-transcriptional gene regulation. FEBS Lett.

[49] Haimovich G (2013). Gene expression is circular: factors for mRNA degradation also foster mRNA synthesis. Cell.

[50] Perez-Ortin JE, de Miguel-Jimenez L, Chavez S (2012). Genome-wide studies of mRNA synthesis and degradation in eukaryotes. Biochim Biophys Acta.

[51] Mitrovich QM, Anderson P (2005). mRNA surveillance of expressed pseudogenes in C. elegans. Curr Biol.

[52] He F (2003). Genome-wide analysis of mRNAs regulated by the nonsense-mediated and 5′ to 3′ mRNA decay pathways in yeast. Mol Cell.

[53] Sayani S, Janis M, Lee CY, Toesca I, Chanfreau GF (2008). Widespread impact of nonsense-mediated mRNA decay on the yeast intronome. Mol Cell.

[54] Hansen KD (2009). Genome-wide identification of alternative splice forms down-regulated by nonsense-mediated mRNA decay in Drosophila. PLoS Genet.

