# Supplementary material for: PARP1 is a versatile factor in the regulation of mRNA stability and decay
Source: Sci Rep. 2019 Mar 6;9:3722. doi: 10.1038/s41598-019-39969-7 (PMC6403249; doi:10.1038/s41598-019-39969-7)
Supplement: Supplementary file 1 — Supplementary Figures and Tables [file 41598_2019_39969_MOESM1_ESM.docx]

**PARP1 is a versatile factor in the regulation of mRNA stability and decay**

**Matveeva A. Elena^1^, Lein F. Mathbout^1,2^ and Yvonne N. Fondufe-Mittendorf^1^***

*^1^Department of Molecular and Cellular Biochemistry, University of Kentucky, Lexington, KY USA*

*^2^College of Medicine, Alfaisal University, Al Maather’ Riyadh, Saudi Arabia*

# *Corresponding author:

Yvonne N. Fondufe-Mittendorf

741 S. Limestone, 273 BBSRB

University of Kentucky

Lexington, KY 40536

Email: y.fondufe-mittendorf@uky.edu

# Other author email addresses:

MAE: elmatva@email.uky.edu

LFM: lmathbout@alfaisal.edu

# Competing interests

The author(s) declare no competing interests.

**Supplementary Figures and Tables**


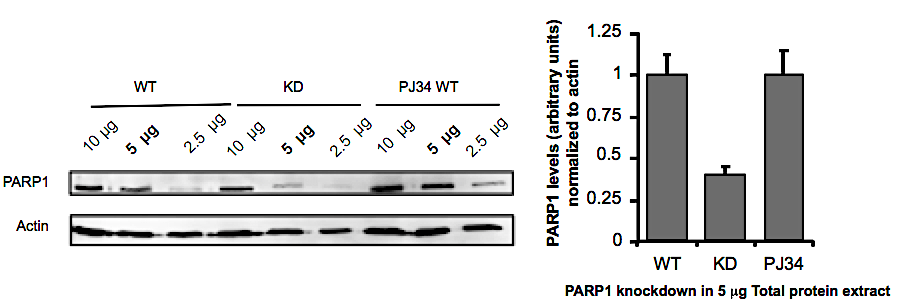


**Suppl. Fig. S1. Measurement of PARP1 levels in WT, KD and PARylation inhibited cells.** Serial dilution of the total protein extracts from experimental samples (WT, PARP1 KD and PARylation inhibited cells), were run on a PAGE gel. Left is a representative western blot analysis of PARP1 levels in experimental samples and right is the quantification of PARP1 levels from the 5 μg protein extract.


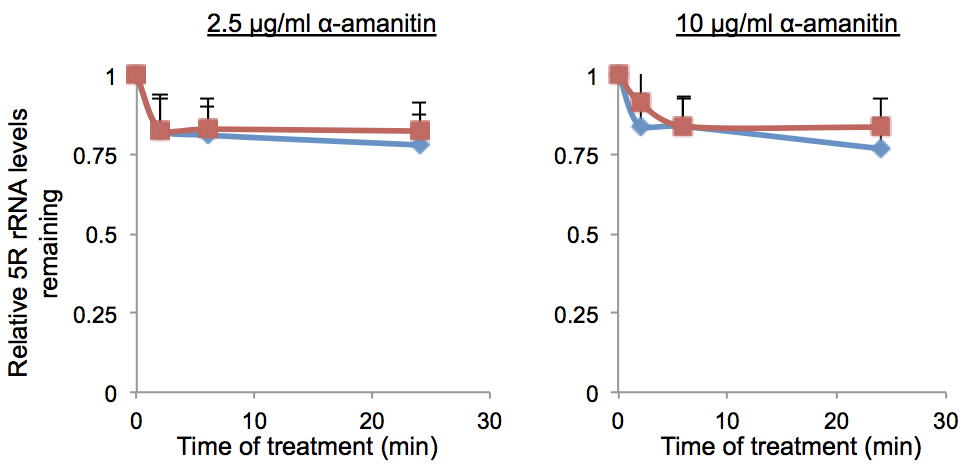


**Suppl. Fig. S2. PARP1 has no effect on 5sRNA mRNA stability after α-amanitin treatment under experimental concentrations.** Half-lives of 5S rRNA, were measured using qRT-PCR in WT (blue) and KD (red) cells. The decay rates were measured post α-amanitin treatments. Results are also represented as mean ± SEM of three independent experiments. All results are significant as analyzed by student T-test p<0.05.

**
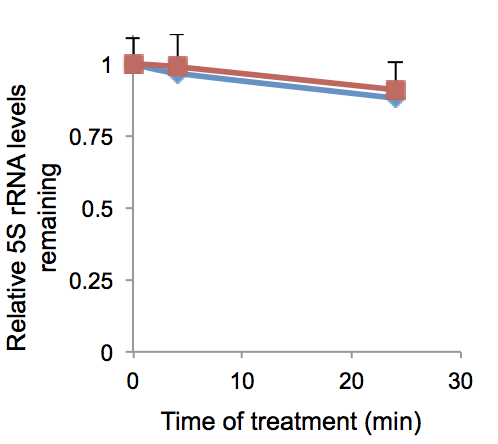
**

**Suppl. Fig. S3. PARP1 has no effect on 5sRNA stability and expression after DRB treatment.** Measurement of 5sRNA stability after DRB treatment.

**Suppl. Fig. S4.** **Treatment of WT and PARP1 KD cells confirms the destabilization effect of PARP1 after DRB treatment**. Half-lives of mature *AKAP200* mRNA (exon-exon primers) were measured using quantitative RT-PCR in WT and PARP1 KD cells following transcription inhibition by DRB treatment. Results are shown as mean ± SEM from three independent experiments. Comparative analyses between WT and KD, were significant as analyzed using student T-test p<0.05


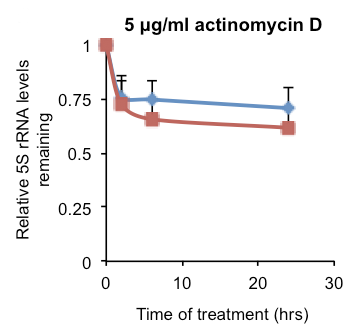


**Suppl. Fig. S5. NPARP1 has no effect on 5s rRNA stability after actinomycin D treatment.** qRT-PCR was used to measure 5s rRNA stability after actinomycin D, which also targets RNAPIII transcription. PARP1 has a negligible effect on 5S rRNA stability after actinomycin D treatment.

**
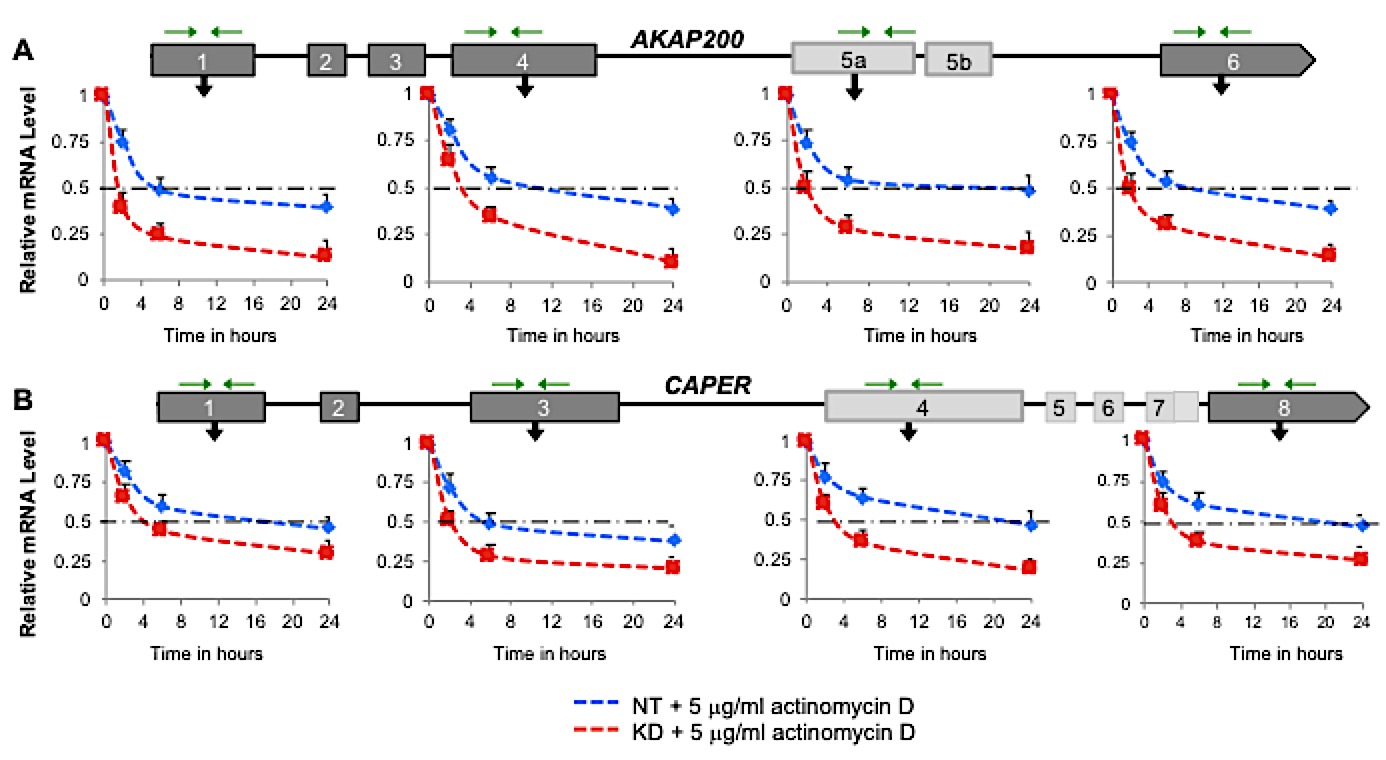
**

**Suppl. Fig. S6.** **Treatment of WT and PARP1 KD cells confirms the destabilization effect of PARP1 after actinomycin D treatment.** Half-lives of *AKAP200* (A) and *CAPER* (B), were measured using qRT-PCR in WT and PARP1-KD cells**.** S2 Drosophila cells were transfected with siCon and siPARP1. The decay rates of total mRNAs were assessed using quantitative RT-PCR in WT and PARP1 KD cells following transcription inhibition by actinomycin D treatment. Results are shown as mean ± SEM from three independent experiments. Comparative analyses between WT and KD, were significant as analyzed using student T-test p<0.05.

**Suppl. Table 1**: Half-lives in hours of *AKAP200 and CAPER* mRNAs as measured from the different exons after actinomycin D treatment.

| Gene/Exon | actinomycin D | actinomycin D |
| --- | --- | --- |
| ***AKAP200*** | **WT** | **KD** |
| 1 | 5.8 | 1.6 |
| 4 | 11.8 | 3.9 |
| 5 | 19.8 | 2 |
| 6 | 10.5 | 2 |
| ***CAPER*** | **WT** | **KD** |
| 1 | 18.5 | 4.8 |
| 3 | 5.8 | 2 |
| 4 | 18.5 | 4.8 |
| 8 | 20.3 | 2.5 |


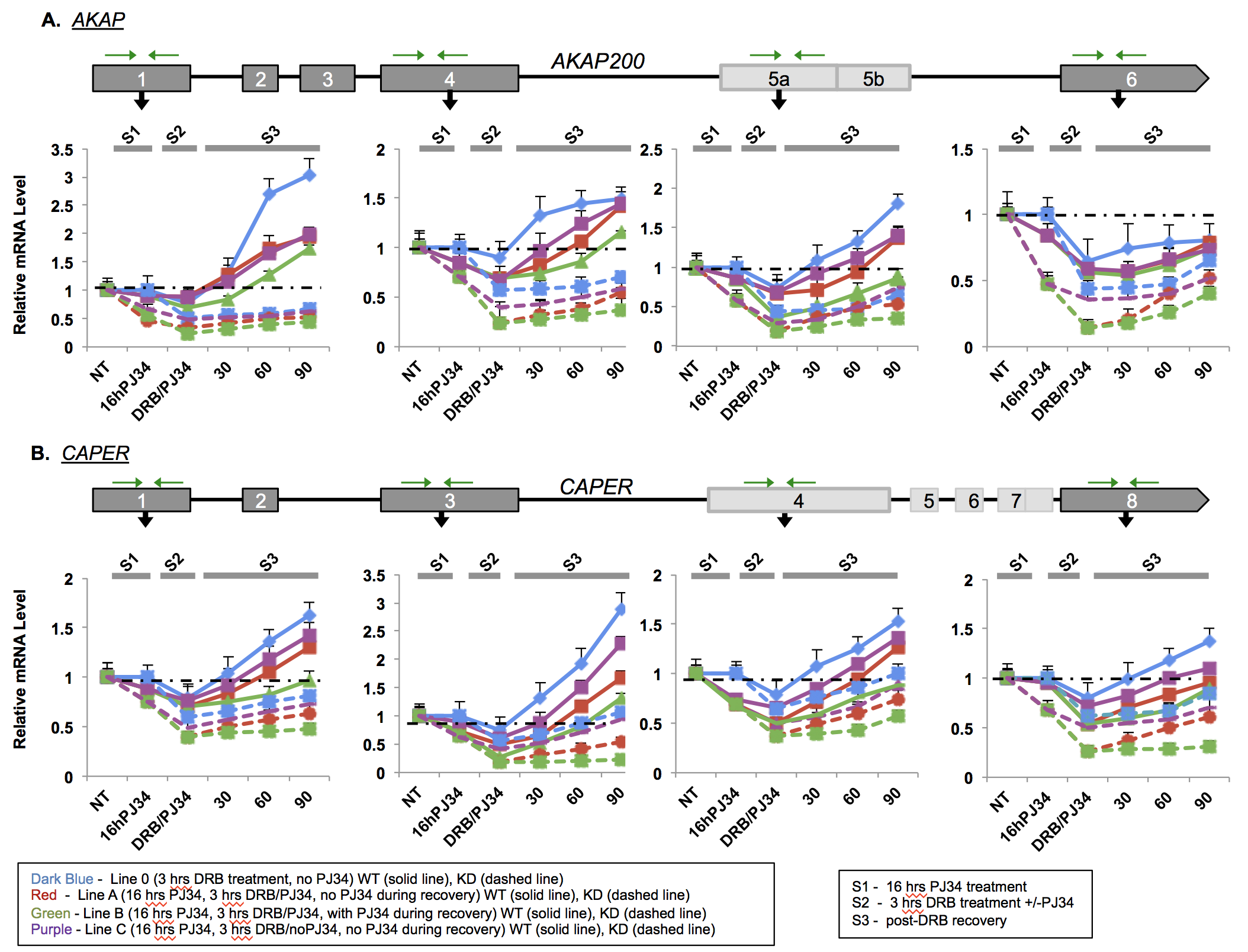


**Suppl. Fig. S7**: **Direct comparison of PARP1 and PARylation effects on total mRNA levels in WT and KD cells**. The results represented in this figure are same with Fig. 3 and represents direct comparison on the same graph. Total mRNA levels using exonic primers (green arrows) were measured at each stage of the experiment. WT for and PARP1 KD cells were treated with PJ34 overnight, to inhibit PARylation. Control cells are cells in each condition (WT or KD) that was not treatment with PJ34 at each stage. At the beginning (Stage 1 or S1), mRNA levels were measured from cells. Next, cells were incubated with 30 μg/ml DRB for 3 hrs (stage 1 or S1); then DRB containing medium was removed, fresh medium was added and transcription was allowed to resume (stage 3 or S3). At the stage of recovery (S3), levels of total RNA as measured by RT-PCR using exonic primers (green arrows) were determined for *AKAP200* **(A)** and *CAPER* **(B)**. mRNA levels were normalized to the values prior to PJ34 treatment sample, which was set to 1. Results are shown as mean ± SEM from three independent experiments. All results were measured in relation to the non-treated cells in each condition (WT or KD) and results were significant as analyzed using student T-test p<0.05.


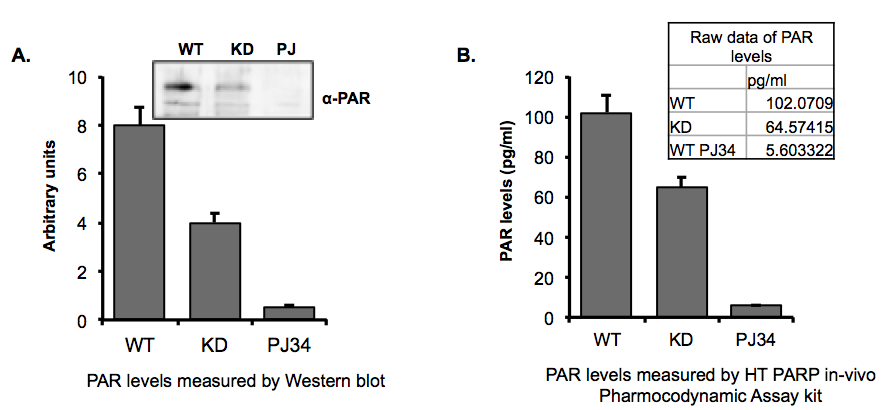


**Suppl. Fig. S8. Measurement of PAR levels in WT, KD and PJ34 (PARylation inhibited) treated cells**. **(A)** Samples were also analyzed using antibody against PAR. Quantification of PAR levels from these western blot analyses (inset from 5 μg total protein extracts), were used to quantify the levels of PAR in these cells. **(B)** PAR levels were also measured using a pharmacodynamics Assay kit. Insert is a table of the normalized numbers used to draw the bar chart.

### Supplementary Table S2: List of primers used in the study

| Gene | # | Primer name | Direction | Primer sequence (5'-3') |
| --- | --- | --- | --- | --- |
| PARP1 | 1 | PARP1/F | Forward | TCGACGTGTCGTGGATGTGAACAA |
| PARP1 | 2 | PARP1/R | Reverse | ACAAAGGTTGGCCTCCGTACTTCA |
| Actin | 3 | Actin5C/F | Forward | TCGCGATTTGACCGACTACCTGAT |
| Actin | 4 | Actin5C/R | Reverse | TTGATGTCACGGACGATTTCACGC |
| AKAP | 5 | Ex1/F | Forward | ACATCCTAACGCGACGTAAATA |
| AKAP | 6 | Ex1/R | Reverse | CTGCTTTCCGTTTCGGTTTC |
| AKAP | 7 | Ex4/F | Forward | CTGCTGCTGGTGAGGATATAA |
| AKAP | 8 | Ex4/R | Reverse | GTCCTTCTTGCCAAAGGAAATG |
| AKAP | 9 | Ex5/F | Forward | AGTTGAAGCCAAGTCCGTAG |
| AKAP | 10 | Ex5/R | Reverse | TCCACAATAACGGACTCGAAC |
| AKAP | 11 | Ex6/F | Forward | GATCTCGCCAAGGATCTGAA |
| AKAP | 12 | Ex6/R | Reverse | GAGTAGGATTATTCGCATGTAACG |
| AKAP | 13 | Ex1/Intr1/F | Forward | CCAGTGTAGAATCGCAAAGC |
| AKAP | 14 | Ex1/Intr1/R | Reverse | AGTGGAATTTACCTGGCTCG |
| AKAP | 15 | Ex4/Intr4/F | Forward | CTGTCGCAGAACCATCGG |
| AKAP | 16 | Ex4/Intr4/R | Reverse | ATTTCGAGTGTCAACTTACCTCA |
| AKAP | 17 | Ex5/Intr5/F | Forward | TGCTGGTAGAATTGGATGCG |
| AKAP | 18 | Ex5/Intr5/R | Reverse | CGATGTGTTTTGAAGACTTGAGG |
| AKAP | 19 | Intr5/Ex6F | Forward | GCAAAAGCCTTGACCAGATAAAC |
| AKAP | 20 | Intr5/Ex6F | Reverse | CTTCTCCTTCAGATCCTTGGC |
| CAPER | 21 | Ex1/F | Forward | TCGATAAGTTCTGTGACAACCC |
| CAPER | 22 | Ex1/R | Reverse | CGCACAGCGGAATTCGTA |
| CAPER | 23 | Ex3/F | Forward | CCCGAATTGCAGCGAAGTA |
| CAPER | 24 | Ex3/R | Reverse | TTATCCGGCCTTTGGGAAC |
| CAPER | 25 | Ex4/F | Forward | TGTCTTTGCAGAGAATCGAATAAG |
| CAPER | 26 | Ex4/R | Reverse | CAGCTTCTGGCACATTCAAA |
| CAPER | 27 | Ex8/F | Forward | GTGGACACGATGACGACTAC |
| CAPER | 28 | Ex8/R | Reverse | GGGATCGTTTGCTTTGCTT |
| CAPER | 29 | Ex1/Intr1/F | Forward | ACTTTGATGTGGAGGCGATG |
| CAPER | 30 | Ex1/Intr1/R | Reverse | ACGATAGTTGGCTGTTATCCG |
| CAPER | 31 | Ex3/Inrtr3/F | Forward | TTTTCCCACATAGACACCCAC |
| CAPER | 32 | Ex3/Intr3/R | Reverse | ACCACTTAGTATGCATCGACTG |
| CAPER | 33 | Ex4/Intr4/F | Forward | TTGCTAGAGATTGTTTTGGTCATTC |
| CAPER | 34 | Ex4/Intr4/R | Reverse | AATGCCTAGATATGTTTTGGTTTTTTT |
| CAPER | 35 | Intr8/Ex8/F | Forward | ACCCATATCCTCCCTGTCC |
| CAPER | 36 | Intr7/Ex8/R | Reverse | CCCGCACTGACATTCTACAC |
| AKAP | 37 | Ex2/Ex3 | Forward | GAAAGAAGCAAGTGCGTGTG |
| AKAP | 38 | Ex2/Ex3 | Reverse | AGAACCGTTAAAGTCGAGCG |
| AKAP | 39 | Ex3/Ex4 | Forward | AGTTTTCACCTAGTTAAGAGAGATAGAA |
| AKAP | 40 | Ex3/Ex4 | Reverse | TGGTCAGATCTTTGTCGTTCTC |
| AKAP | 41 | Ex4/Ex5 | Forward | CAAATGGCGAGGCTGAAAAG |
| AKAP | 42 | Ex4/Ex5 | Reverse | GGCTTCTCATCCTTGGACG |
| AKAP | 43 | Ex5/Ex6 | Forward | TGCTGGTAGAATTGGATGCG |
| AKAP | 44 | Ex5/Ex6 | Reverse | CTTCTCCTTCAGATCCTTGGC |


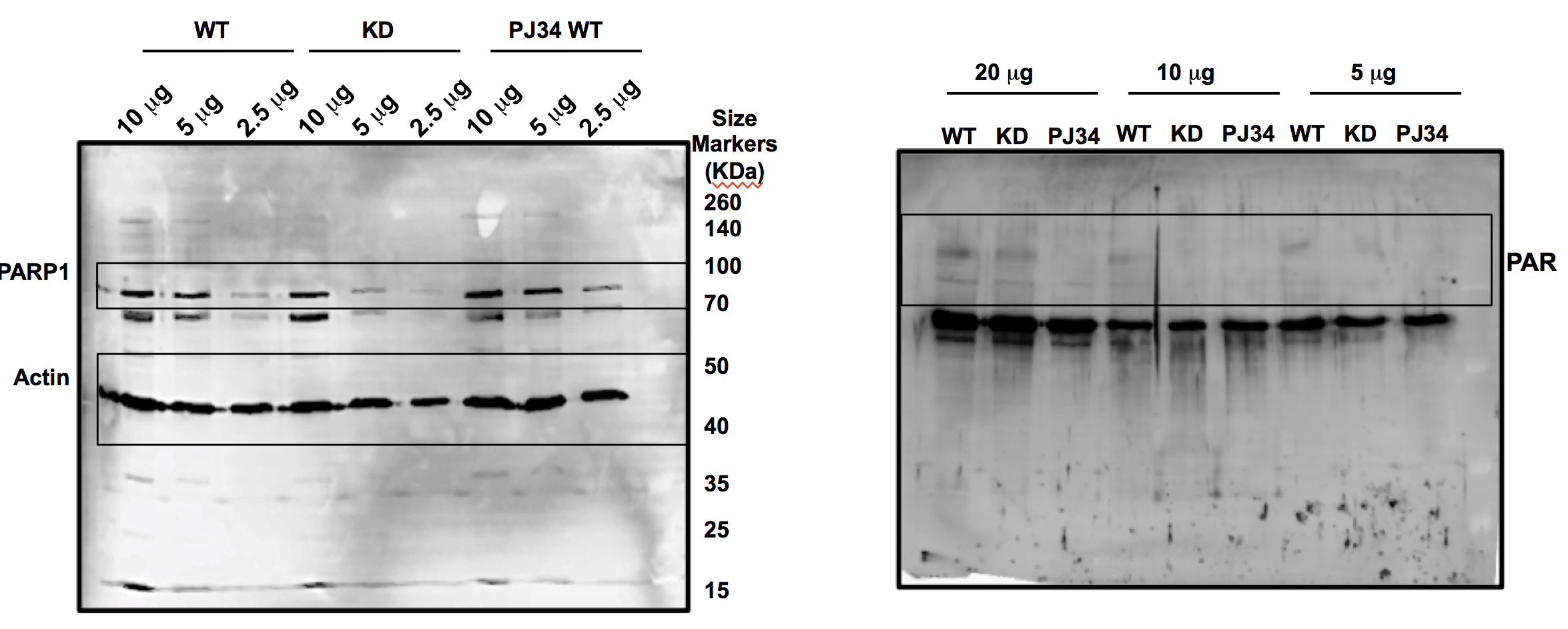


**Full gel image of the western blot analyses in Suppl. Fig. 1 and 8**
